# Identification of *Bacillus anthracis* Strains from Animal Cases in Ethiopia and Genetic Characterization by Whole-Genome Sequencing

**DOI:** 10.3390/pathogens14010039

**Published:** 2025-01-07

**Authors:** Abebe Olani, Domenico Galante, Matios Lakew, Bayeta Senbeta Wakjira, Getnet Abie Mekonnen, Tesfaye Rufael, Tsegaye Teklemariam, Wondwosen Kumilachew, Shimalis Dejene, Ayele Woldemeskel, Adanech Wakjira, Getachew Abichu, Baye Ashenafi, Nigatu Kebede, Aklilu Feleke Haile, Fufa Dawo Bari, Laura Del Sambro, Tadesse Eguale

**Affiliations:** 1 Animal Health Institute, Sebeta P.O. Box 04, Ethiopia; matioslakew@gmail.com (M.L.); didigabruma@gmail.com (B.S.W.); getnet.abie.mekonnen@gmail.com (G.A.M.); chibssasafo@gmail.com (T.R.); gechsor1992@yahoo.com (G.A.); 2 Aklilu Lemma Institute of Pathobiology, Addis Ababa University, Addis Ababa P.O. Box 1176, Ethiopia; nigatu.kebede@aau.edu.et (N.K.); aklilu.feleke@aau.edu.et (A.F.H.); tadesse.eguale@aau.edu.et (T.E.); 3 Istituto Zooprofilattico Sperimentale della Puglia e della Basilicata, Anthrax Reference Institute of Italy, 71121 Foggia, Italy; laura.delsambro@izspb.it; 4 Mizan Veterinary Laboratory Center, South West Ethiopia Region, Mizan P.O. Box 251, Ethiopia; tsege2004@gmail.com (T.T.); ayelewoldemeskel2@gmail.com (A.W.); 5 Bahir-Dar Regional Veterinary Laboratory, Bahir-Dar P.O. Box 70, Ethiopia; wonduzikumilachew@gmail.com; 6 Sodo Regional Veterinary Laboratory, Sodo P.O. Box 82, Ethiopia; shimdejene21@gmail.com; 7 Negelle Health Science College, Negelle P.O. Box 186, Ethiopia; adiwkjira@gmail.com; 8 Department of Public Health, Institute of Public Health, University of Gondar, Gondar P.O. Box 196, Ethiopia; bayewassieashenefe@gmail.com; 9 Department of Microbiology, Immunology and Veterinary Public Health, College of Veterinary Medicine and Agriculture, Addis Ababa University, Bishoftu P.O. Box 1176, Ethiopia; fufa.bari15@gmail.com; 10 Ohio State Global One Health, Addis Ababa, Ethiopia

**Keywords:** *Bacillus anthracis*, epidemiology, Ethiopia, molecular characterization, whole genome sequencing

## Abstract

Anthrax is a zoonotic disease characterized by rapid onset with usual fatal outcomes in livestock and wildlife. In Ethiopia, anthrax is a persistent disease; however, there are limited data on the isolation and molecular characterization of *Bacillus anthracis* strains. This study aimed to characterize *B. anthracis* isolated from animal anthrax outbreaks between 2019 and 2024, from different localities in Ethiopia. *B. anthracis* was identified using standard microbiology techniques and confirmed by real-time PCR. For the first time in Ethiopia, the genetic diversity of five *Bacillus anthracis* strains, isolated from dead cattle and goats, was investigated by Whole Genome Sequencing (WGS) and bioinformatics analyses. The five sequenced strains were compared to one Ethiopian *B. anthracis* genome and the other 29 *B. anthracis* genomes available in the global genetic databases to determine their phylogeny. The genomes of the strains were also analyzed to detect the presence of antimicrobial resistance and virulence genes. The whole genome SNP analysis showed that the Ethiopian *B. anthracis* strains were grouped in the A clade. Three strains (BA2, BA5, and BA6) belonged to the A.Br.034 subgroup (A.Br.005/006), and two strains (BA1 and BA4) belonged to the A.Br.161 (Heroin) clade of the Trans-Eurasian (TEA) group. The findings of this study will contribute to expanding the current understanding of the anthrax hotspots in Ethiopia, and the phylogenetic correlation and/or diversity of the circulating strains.

## 1. Introduction

*Bacillus anthracis* is a Gram-positive, spore-forming, rod-shaped bacterium and the causative agent of anthrax in humans, livestock, and wildlife worldwide. Anthrax is classified as a neglected and underreported zoonosis [1], a group of diseases shared by animals and people that receive less than 0.1% of international global health assistance due to their occurrence in remote, disadvantaged communities [2]. Annually, an estimated 20,000 to 100,000 cases of anthrax occur worldwide, predominantly in poor rural areas [3]. The disease remains endemic in many low and middle-income countries [4] and more rarely occurs in Europe, due to strict prophylactic measures [5,6,7].

*Bacillus anthracis* remains dormant in the soil as a spore. These spores can resurface after heavy rain [8] or as a result of disturbances to animal burial sites [9], leading to outbreaks [10]. Animals become infected by ingesting spores from the soil while grazing. Once inside the host, the spores germinate, producing toxin-producing, encapsulated bacilli. The anthrax pathogen is known for unexpected re-emergence after years or decades of inactivity at previous outbreak sites [8].

*Bacillus anthracis* is one of the most genetically homogeneous bacteria. Molecular characterization of *B. anthracis* is valuable for epidemiologic and epizootiologic studies, allowing for the monitoring of the spread and distribution of anthrax outbreaks [9]. The current genotyping methods for *B. anthracis* include the analysis of 13 stable and non-homoplastic loci known as canonical single-nucleotide polymorphisms (SNPs), multi-locus variable number of tandem repeats analysis (MLVA) [11], whole genome sequencing (WGS) [12], and the cgMLST [13].

A WGS approach provides better diagnostic resolution power to differentiate lineages by identifying thousands of single nucleotide polymorphisms (SNPs) retrieved from aligned sequences of *B. anthracis* genomes [14,15] and is a powerful alternative to looking at disease outbreak origins, introductions, and routine surveillance to support epidemiological investigations [12,16]. The global population structure of *B. anthracis* is divided into three major canonical clades, namely the A, B, and C branches [17]. These groups are subdivided into 13 distinct lineages, robustly defined by key point mutations (canSNPs) [18].

Anthrax is an endemic disease and ranks second, next to rabies, as the disease of greatest zoonotic concern in Ethiopia [19], with sporadic outbreaks typically occurring in May and June [20]. Anthrax outbreaks have been reported in domestic ruminants and humans in Amhara, Oromia, Tigray, and the Southern region of Ethiopia [21]. A retrospective study by Bahiru et al. [21] showed that a total of 5197 human cases, 86 deaths, 26,737 animal cases, and 8523 deaths were documented from 2009 to 2013 nationally. In Ethiopia, humans are at risk of infection mainly due to the tradition of consuming raw meat, using hides for different purposes, and sharing home-butchered meat among the community, resulting in the exposure of several people to contaminated meat from a single anthrax case [22].

The prevention and control of anthrax in Ethiopia are constrained by several factors, including a poor understanding of the disease dynamics, weak infrastructure, and high-risk sociocultural practices. Reporting of cases and outbreaks is sporadic, and there is little knowledge of hotspots and almost no understanding of the dynamics of infection and circulating genotype(s) in the country. There is limited information available on the identification and characterization of *B. anthracis*, except for a few reports on culture-based identification [23] and a single report of PCR detection in humans [24].

Only a few partial or draft genome sequences of *B. anthracis* strains from Ethiopia are available in public databases. Out of these, only one sequence has its sequence files deposited in the SRA databases, enabling comparative analysis [25,26]. Hence, there is an urgent need to better understand the extent of genetic diversity among *B. anthracis* strains in Ethiopia. Due to a lack of knowledge about the risks associated with the improper removal of infected carcasses, the disease is not properly monitored, and socio-economic conditions contribute to the underreporting and underdiagnosis of anthrax in Ethiopia.

Genomics-based molecular characterization and strain differentiation of *B. anthracis* allow for the identification of outbreak sources based on host and geography. The ability to rapidly assess and accurately detect *B. anthracis* infection/exposure and share sample confirmation is a cornerstone of anthrax control, prevention, and elimination. Hence, the purpose of this study is to address the limited information on the isolation, identification, and circulation of *B. anthracis* in Ethiopia, and to assess the extent of genetic diversity among these strains in the country, filling the gap in underreporting and underrepresentation within the global community.

## 2. Materials and Methods

### 2.1. Study Area

**Figure 1:**
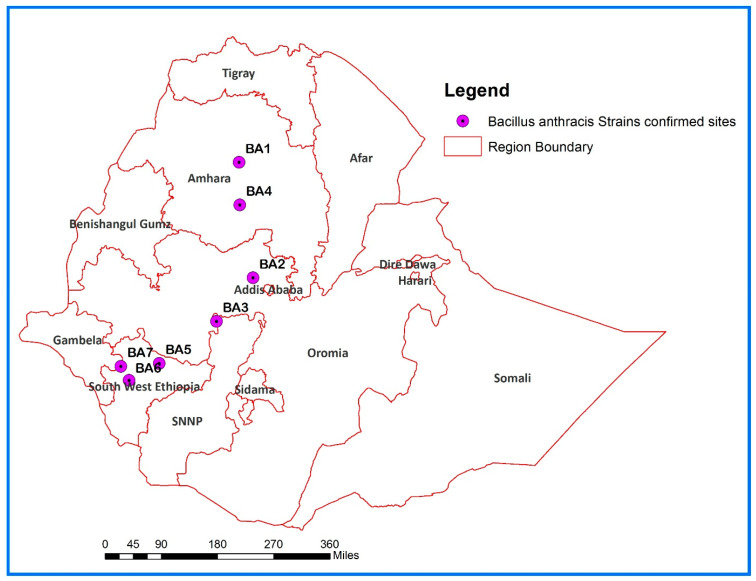
Map of Ethiopia showing the sites where *B. anthracis* strains were isolated.

This study was conducted in different parts of the country to investigate reported animal anthrax outbreaks between 2019 and 2024 in four regions of Ethiopia (Figure 1). Specifically, seven animal anthrax outbreaks were investigated in the following locations: Gurage Zone (Abeshge District), Central Ethiopia Region; West Shoa Zone (Ada Berga District), Oromia Region; Kefa Zone (Bonga Town); Bench Sheko Zone (South-Bench District); Sheka Zone (Yeki District) of South West Ethiopia Region; and South Gondar (Farta District) and East Gojjam (Goncha Siso Enese) of Amhara Region.

### 2.2. Anthrax Outbreak Investigation

Samples were collected from carcasses in accordance with the multi-sectoral zoonotic disease outbreak investigation guidelines [27]. This investigation was part of the routine disease monitoring activities conducted by the National Animal Health Institute, prompted by reports from local animal health offices in regions or zones where suspected anthrax cases were identified. The sources of samples for the outbreak investigation included domestic animals (cattle and goats), following reports of suspected anthrax cases. The purposeful collection included tissue samples from dead animals (fresh and old carcasses) suspected of having anthrax: ear clips, eyelids, swabs (nasal, rectal, blood and bone) and dried meat. 

### 2.3. Sample Collection and Transportation

#### Samples Collected from Suspected Animal Anthrax Outbreaks

The approach for collecting samples from suspected animal anthrax outbreaks was based on the type of specimen to examine. Samples were collected from fresh and old animal carcasses/products [8]. Archived samples collected from suspected animal anthrax outbreaks from 2019 to 2024 at the Animal Health Institute (AHI) were also used. The source of specimens, place of collection, and type of specimens used to isolate *B. anthracis* are listed in Table 1. Swabs from oozing blood and old bone, clippings of the ears and eyelids were taken from fresh carcasses. Additionally, any bloody materials, dried meat, and visible blood under the head or the tail of dead animals were also collected.

The sampling date, sample type, GEO references (latitude, longitude, and altitude), and sample identification were clearly labeled and transported in accordance with the International Air Transport Association (IATA) packing instructions for biological samples. Tissue samples, including ear and eyelid clippings, were transported under cold chain conditions. In contrast, swabs containing oozing blood, bloody materials, and dried meat were transported at room temperature. All samples were securely packed using triple packaging and stored at 4 °C until processed at the Animal Health Institute (AHI) in Sebeta, Ethiopia, for identification and molecular detection, as previously described [28]. All samples were handled in a biosafety level 3 (BSL-3) laboratory at the Animal Health Institute, Sebeta, Ethiopia.

### 2.4. Isolation and Identification of B. anthracis

#### 2.4.1. Identification Using Gram-Staining and Capsule Staining

Smears were prepared from ear clips, and nasal and anal blood discharges, as well as culture isolates. Gram staining was performed to reveal the presence of Gram-positive, straight bacilli. These bacilli usually appear singly, in pairs, or in short chains of three to four cells [8]. The capsule was induced by growing on nutrient agar (Oxoid, Basingstoke Hampshire, UK) containing 0.7% sodium bicarbonate (Sigma-Aldrich, St. Louis, Missouri, USA) and incubated at 37 °C for 24 h. Smears were prepared from cultured isolates of dead animals to detect the capsule of *B. anthracis*. Bacteria were looked for in smears of these specimens that have been dried, fixed either using heat or by dipping the smear in 95–100% alcohol for about 1 min, and air-dried. Then, stained with polychrome methylene blue (M’Fadyean reaction), allowed to dry, and examined under the 10× lens and oil immersion. The capsule stains are pink, whereas the bacillus cell stains are dark blue [8].

#### 2.4.2. Culture

Swabs containing oozing blood and bone were cultured on Columbia agar (Oxoid, Basingstoke Hampshire, UK) with 5% sheep blood at a temperature of 35–37 °C for 18–24 h [8]. Following incubation, the growth was characterized by colony morphology; *B. anthracis* colonies appear as gray/white, flat colonies measuring 2–5 mm in diameter with irregular edges. The haemolytic patterns of the bacteria were assessed based on the types of haemolysis observed on Columbia agar (Oxoid, Basingstoke Hampshire, UK) [29]. Pure colonies were preserved in sterile 20% glycerol brain heart infusion broth (HiMedia, Mumbai, Maharashtra, India) and stored at −20 °C until further testing. Sample processing, isolation, and inactivation of the isolate were carried out at the Animal Health Institute (AHI) in a biosafety level 3 laboratory using a class II type A2 biosafety cabinet.

#### 2.4.3. Biochemical Test

The motility test was performed using a motility test medium (HiMedia, Mumbai, Maharashtra, India) with triphenyl tetrazolium chloride (Sigma-Aldrich, St. Louis, Missouri, USA) and *B. anthracis* suspected culture isolates were stab-inoculated using a straight wire down the middle of the motility medium (semi-solid). Penicillin G sensitivity tests of isolated *B. anthracis* colonies were performed on Muller–Hinton agar (Oxoid, Basingstoke Hampshire, UK) using the disk diffusion method [8]. Unlike *B. cereus* species, *B. anthracis* is non-motile and susceptible to penicillin.

### 2.5. Molecular Detection and Characterization

#### 2.5.1. Growth of *B. anthracis* and DNA Extraction

Pure colonies of *B. anthracis* strains collected from outbreaks (Table 1) were grown on Columbia agar (Oxoid, Basingstoke Hampshire, UK) at 37 °C for 18 h. The colony was harvested and digested into 100 µL of nuclease-free water in a 1.5 mL microcentrifuge tube. Next, 100 µL of lysis buffer (20 mM Tris HCI, Triton 1.2%, EDTA, and lysozyme (Sigma-Aldrich, St. Louis, Missouri, USA) 80 mg per 1 mL buffer solution) was added and incubated at 37 °Cfor 1 h. Genomic DNA extraction was performed using the DNeasy Blood and Tissue kit (Qiagen, Hilden, Germany), following the extraction instructions for Gram-positive bacteria [30,31]. To remove any potential spore contamination, the DNA extract was filtered through 0.1 μm Ultrafree-MC filter units [32].

The sterility of purified DNA was confirmed by cultivating 20 μL of each on Columbia agar, then incubating at 37 °C for 72 h. Further DNA processing was conducted only with negative results [25]. DNA concentrations were quantified using the UV/Vis Nano Spectrophotometer (Neo Biotech, Sinpyeongro, Republic of Korea) according to the supplier’s protocol. The DNA sample was considered sufficiently pure if it had an absorption coefficient of 260/280 in the range of 1.7–2.0 [33]. DNA was stored at −20 °C until further use.

#### 2.5.2. Diagnostic Real-Time PCR for Detecting Plasmid Markers of *B. anthracis*

The presence of specific virulence factors for anthrax toxin *pag*A encoded by genetic marker carried on plasmid pXO1 and anthrax capsule *caps* carried on the plasmid pXO2 were detected using real-time PCR [34]. Target primers and probes used to detect *B. anthracis* by real-time PCR are shown in Table 2.

A real-time PCR reaction consisting of 10 µL of Luna Universal Probe qPCR Master mix (New England BioLabs, Hertfordshire, UK) was run using a QuantStudio™ 6 (Thermo Fisher Scientific, Waltham, Massachusetts, USA). Five µL of DNA template, and five of water, as (negative control) were added to obtain a 20 µL final volume.

Template DNA was initially denatured by heating at 95 °C for 1 min. This was followed by 40 cycles of denaturation at 95 °C for 15 s and primer extension at 60 °C for 30 s. A positive control of pLepBaBP+ plasmid DNA (a sample of known DNA) was used, which contains the target of interest instead of a virulent strain, allowing for positive control without culture. Real-time PCR results were interpreted as positive when the fluorescence signals of the *pagA* and *capC* genes showed amplification curves within less than 40 cycles, and negative when the signals exhibited amplification curves beyond 40 cycles [34].

#### 2.5.3. Whole Genome Sequencing

WGS of *B. anthracis* strains was performed at the Istituto Zooprofilattico Sperimentale della Puglia e della Basilicata, Italy. For this aim an aliquot of DNA for each positive sample was sent ensuring a suitable storage temperature. Paired-end genomic libraries were prepared using the Illumina DNA Prep Illumina (Illumina, San Diego, CA, USA). Sequencing was performed on the Illumina MiSeq platform with 500-cycle chemistry as previously reported [35]. High-quality paired-end reads (Q ≥ 30) were performed using Shovill, which utilizes SPAdes (Version 3.15.5) as the genome assembler with the “only assembler” option [36] to create draft genomes. Genome assemblies were evaluated using QUAST (Quality Assessment Tool) [37]. Pilon (version 1.22) [38] was used for correcting SNPs or closing small gaps and INDELs. The obtained scaffolds were manually checked for contaminant reads and annotated automatically by the National Center for Biotechnology Information (NCBI) Prokaryotic Genome Annotation Pipeline [39].

The analysis of all paired-end sequencing data were automated using pipeline workflows within Galaxy [40], with the data available upon request via GitLab. For scalability and efficient run-time, the pipeline was implemented using the Snakemake workflow management system [41]. The pipeline includes steps for quality control of all samples using FASTQC [42], calculation of theoretical genome coverage, and taxonomic classification of the reads using Kraken 2 [43]. Virulence factors were predicted based on the genome assemblies using BTyper (version 3.4.0) [44] against the Virulence Factor Database [45,46] pipeline. Antimicrobial resistance genes were identified using NCBI AMRFinder (version 3.11.26) with the BLAST tool, applying thresholds of 80% coverage and 75% identity against the NCBI database [47].

The identified SNPs were extracted from the software package using bcftools mpileup (version 1.17) and saved as a Virtual Card Format (VCF) file. To improve the quality of the data, SNPs that were less than 10 bp apart or contained unspecified nucleotides (“N”) were removed. Custom Python scripts were employed to search for specific SNPs in the filtered VCF file, which only contained high-quality SNPs. A SNP was considered specific if it was present in all strains of a subclade and absent in all other strains included in the study. The edited file was then used as input in bcftools to generate a FASTA file [25].

#### 2.5.4. Comparison with Public Database Entries

To compare the strains with the global population of *B. anthracis*, we selected entries from the public repositories Sequence Read Archive (SRA) and NCBI GenBank as of August 2024. These entries were processed using established methods [18]. We chose the entries based on their affiliation with various canSNP groups, as outlined previously [18], and their geographic proximity to Ethiopia. To confirm that these strains were indeed *B. anthracis*, we calculated their average nucleotide identity (ANI) to the reference genome *B. anthracis* ‘Ames Ancestor’ (NC_007530.2) using fastANI v1.1 [48]. We applied a threshold of at least 98.9% nucleotide identity for further processing, in consideration of the high genetic stability and correlation of the *B. anthracis* strains. We selected a total of 30 global *B. anthracis* strains, with different subsets being used in the phylogenetic analysis (Table S1).

#### 2.5.5. Phylogenetic Analysis

A whole-genome SNP analysis was conducted using a mapping-based approach with SNP Phylogeny (Samtools) Galaxy (version 24.0) to construct a phylogeny. We compared the five genome sequences with 30 publicly available *B. anthracis* genomes from GenBank (Table S1) and with the reference genome of *B. anthracis* Ames Ancestor (GenBank: NC_007530.2) for the phylogenetic analysis. Genotyping of *B. anthracis* was performed using core-genome multilocus sequence typing (cgMLST) scheme analysis for genetic positioning of the 5 outbreak strains sequenced in this study [13].

The identified SNPs were extracted into a VCF file and converted into a FASTA file, which was used for phylogenetic reconstruction. Phylogenetic trees, along with epidemiological metadata, were visualized using Tree Visualization by One Table (tvBOT) [49] and Grape Tree version 2.6.1 [50].

## 3. Results

### 3.1. Findings from Anthrax Outbreak Investigation

The first suspected outbreak occurred in March 2019 in the Amhara Regional State, South Gondar Zone, Farta District, Wowu kebele. A cow and a goat had died and then a goat was slaughtered, with the meat dried for consumption during the fasting season. There was one human fatality, with symptoms of cutaneous and digestive anthrax preceding death. *B. anthracis* (BA1) was isolated from the dried goat meat.

**Figure 2:**
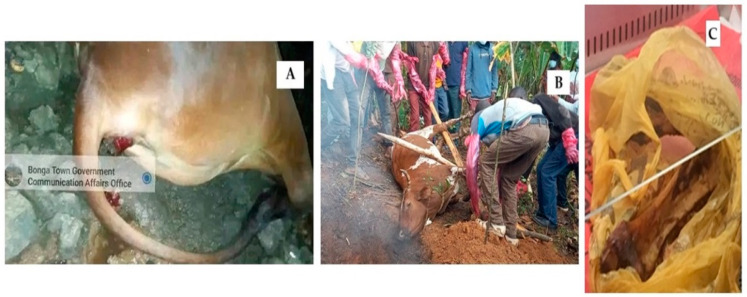
Ox dead of anthrax showing bloody discharge from the anus in Bonga Town (**A**); burying of a cow dead of anthrax in Deber Work Town, 2023 (**B**); bones collected following the death of a cow in Ada Berga District (**C**).

The second anthrax outbreak took place in June 2021 from West Shoa, Ada Berga District, Oromia Regional State, in Bishan Dimo kebele. A total of nine animals died, and one sick cow was slaughtered, with its meat shared among the community, including the owner’s family. All the 21 individuals who consumed the meat became ill with acute gastroenteritis, and two people died. A bone swab was collected from the carcass of a cow (Figure 2C), and *B. anthracis* (BA2) was isolated from the sample.

The third outbreak occurred in December 2021 in Central Ethiopia, Gurage Zone, Abeshege District, Lay Serba kebele, where six goats died, though no human cases were reported. Four samples from one goat (two ear clips and two nasal swabs) were collected, and *B. anthracis* (BA3) was isolated from all samples.

The fourth outbreak took place in Amhara Regional State, East Gojjam, Goncha Siso Enese District, Angot 028 kebele, where three goats died, and three human deaths were reported. A single sample from one goat (skin contaminated with bloody soil) was collected and confirmed positive for *B. anthracis* (BA4).

The fifth outbreak occurred in October 2022 in South West Ethiopia, Kefa Zone, Bonga Town, 02 kebele, where one ox died suddenly during quarantine at the abattoir (Figure 2A). No human cases were reported. A total of four samples (two ear clips and two nasal swabs) were collected, and *B. anthracis* (BA5) was isolated from the tissue samples.

The sixth outbreak took place in March 2023 in South West Ethiopia, Bench Sheko, Debre Work Town, resulting in the death of one cow and seven reported human cutaneous anthrax cases (Figure 2B). Four samples from the cow (two ear clips and two nasal swabs) were collected, and *B. anthracis* (BA6) was isolated from all samples.

The seventh outbreak occurred in February 2024 in the Yeki District, Bench Sheko Zone, South West Ethiopia, where one cow died suddenly with no human cases reported. Four samples (two ear clips and two nasal swabs) were collected, and *B. anthracis* (BA7) was confirmed. In all cases, ring vaccination was carried out, and anthrax outbreaks were limited at all sites.

### 3.2. Identification and Characterization of B. anthracis Strains

**Figure 3:**
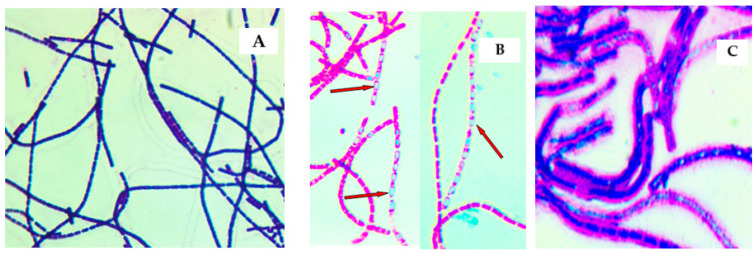
Evidence of Gram-positive, thick long chains of bacilli (**A**); evidence of *B. anthracis* spores (in green) by malachite green staining (**B**); evidence of capsule pinkish-red stained, and bacilli blue stained by Giemsa staining (**C**).

Seven *B. anthracis* strains were collected from 2019 to 2024 (Table 1) and confirmed using the classical microbiology method and real-Time PCR. All *B. anthracis* isolates showed microscopically, typical Gram-positive, thick, long, straight bacilli with square or truncated ends with parallel sides found usually single, in pairs, or long chains of bacilli (Figure 3A). By modified Ziehl–Neelsen and malachite green staining, the presence of spores was also demonstrated (Figure 3B). Mucoid colonies were observed, and Giemsa staining was prepared from bicarbonate agar. The bacilli that were stained appeared blue, square-ended rods in short chains surrounded by a pinkish-red capsule (Figure 3C).

On Columbia agar the colonies appeared non-haemolytic, flat, dry, grayish, and tenacious. The colonies of *B. anthracis* isolates were distinct from other *Bacillus* spp. isolates as they were characterized by a “medusa head”, which appeared with curl-like projections. A real-time PCR test detected the presence of specific virulence factors (*pag*A and *capC)* genes in all the *B. anthracis* isolates tested in this study.

### 3.3. Genetic Characteristics of B. anthracis Strains Isolated from Animals

WGS of the five strains resulted in a total of 8,945,638 reads and the draft genome was composed of 30 to 36 contigs of length >500 bp with an average of N50 contig length of 884,304 bp using SPAdes genome assembly (Table 3). The average total assembly size of the five sequenced strains was 5,548,864 bp representing 99.5% of the reference Ames Ancestor genome (NC_007530.2).

In total, four predicted antimicrobial resistance genes conferring resistance to fosfomycin (*fosB2*), beta-lactam (*bla2* and *bla*), carbapenem, and streptothricin (*satA*), and 16 virulence genes including poly-gamma-glutamate transport (*capA, capB, capC,),* gamma glutamyltranspeptidase required for polyglutamate anchoring to peptidoglycan (*cap),* involved in poly-gamma-glutamate synthesis (*capE*), anthrax lethal toxin *(lef),* calmodulin-sensitive adenylate cyclase edema factor (*cya),* protective antigen *(pagA),* non-hemolytic enterotoxin (*nheA, nheB, nheC*), UTP-glucose-1-phosphate uridylyltransferase (*hasC),* sphingomyelinase (*sph),* phosphatidylinositol-specific phospholipase C (*PI-PLC*), immune inhibitor A metalloproteinase (*inhA*), and layer protein (*bslA*) were detected in all the analyzed strains.

### 3.4. Global Phylogenetic Placement of the Ethiopian B. anthracis Strains

**Figure 4:**
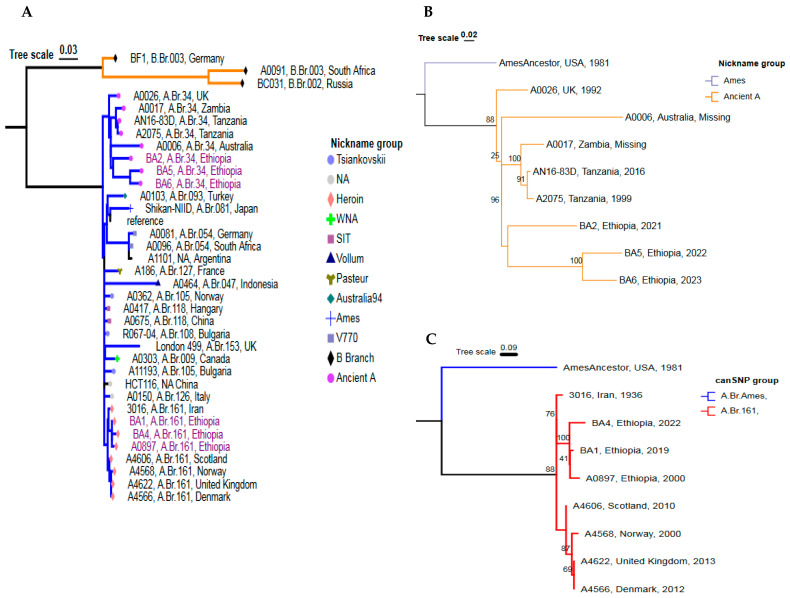
(**A**) Global phylogeny of 35 *B. anthracis* strains generated using the SNP Phylogeny (Samtools) Pipeline, with strains grouped by CanSNP classifications as defined [18]; (**B**) Phylogenetic tree of representatives from the A.Br.034 (Ancient A) CanSNP group, and (**C**) Phylogenetic tree of representatives from the A.Br.161 (Heroin) CanSNP group. The reference genome used was ‘Ames Ancestor’ (NC_007530.2). Colored tips indicate CanSNP group nicknames, while empty circles represent strains that have not yet been assigned to a genetic group.

In this study, the genomic sequence of five outbreak strains and 30 complete *B. anthracis* genomes (1 Ethiopian sequence and 29 global sequences previously published [18]), were used to construct the global phylogeny of the *B. anthracis* strains based on the SNP analysis of the complete genomes. A total of 3036 genome SNPs were identified and used for the construction of a phylogenetic tree via the maximum-likelihood method (Figure 4). Whole genome SNP analysis revealed that the *B. anthracis* strains from Ethiopia were grouped within the A clade but were positioned at two distinct phylogenetic locations. None of the sequenced genomes in this study were grouped under the *B. anthracis* clade B and C. Isolate BA1 and BA4 were isolated from Farta, South Gondar, and Goncha Siso Enese (East Gojjam), respectively, and their genomes are clonal and very closely related to each other.

In this study, two isolated strains collected from Farta, South Gondar (BA1) and Goncha Siso Enese, East Gojjam Zone (BA4) belonged to the Trans-Eurasian (TEA) group, clade A.Br.161 and the other three strains BA2, BA5, and BA6 belonged to A.Br.034 subgroup (A.Br.005/006 sublineage), Ancient A Clade. The TEA A.Br.008/11 sublineage consists of five subgroups “Tsiankovskii”, “SIT”, “Heroin”, “Pasture”, and “Carbosap”. Both BA1 and BA4 strains were very closely related to strain A0897 (Ethiopia), 3016 (Iran), A4606 (Scotland), A4568 (Norway), A4566 (UK), and A4622 (Denmark) which were previously reported [26]. The strain A0897 was separated by a distance of four SNPs from the BA1 strain and eight SNPs from the BA4 strain. BA1 from Farta, South Gondar is closely related to strain BA4 which was isolated in Goncha Siso Enese, East Gojjam Zone of the Amhara Region.

Three Ethiopian strains collected from Ada Berga, West Shoa Zone (BA2), Bonga Town, Kefa Zone (BA5), and Debu-Bench, Bench Sheko Zone (BA6) belonged to the A.Br.034 subgroup (A.Br.005/006 sublineage), Ancient A Clade. Strain BA5 and strain BA6 were closely related to each other and differed, respectively, by 49 SNPs and 48 SNPs from strain BA2. The A.Br.005/006 sublineage (Ancient A Clade) is representative of African strains and is common in Zambia, Tanzania, and South Africa. To this group belong the Tanzanian strain (A2075), the Zambian strain (A0017), but also other non-African strains, such as the Australian strain (A0006), and the UK strain (A0026).

### 3.5. Detailed Genetic Positioning of the 5 Outbreak Strains Using cgMLST Analysis

The results from whole-genome SNP analysis were utilized to construct a minimum spanning tree (MST) for a detailed phylogenetic comparison of the *B. anthracis* strains isolated in this study with the Ames Ancestor strain and the Ethiopian strain (A0897) previously isolated and present in GenBank (Figure S1). Most of the *B. anthracis* strains from Ethiopia were found to belong to the TEA clade A.Br.161 and to the sublineage A.Br.005/006 (Ancient A Clade). The MST indicated limited genetic diversity among the five strains analyzed in this study. The closest strains, BA1 and BA4, belong to the TEA subgroup (A.Br.008/11) and BA1 differs by 21 cgMLST alleles from BA4. Strain BA2 showed 172 different cgMLST alleles from BA6 and 254 allele differences from BA5, both of which are part of the Ancient A Clade, sublineage A.Br.005/006.

## 4. Discussion

In Ethiopia, anthrax is an endemic disease and there is little publicly accessible information on genomic sequences of the isolated stains. This study investigated seven outbreaks, and five genomes of *B. anthracis* strains were sequenced from anthrax outbreaks that occurred in different parts of Ethiopia. WGS was used for the first time, to investigate anthrax outbreaks in animals in Ethiopia, reducing the gap in knowledge about the genetic characteristics of *B. anthracis* strains circulating in this country.

In this study, microbiological methods were used to identify *B. anthracis* and proved to be useful in conjunction with WGS for genotyping purposes [51]. The whole genome analysis revealed the presence of predicted resistant genes against fosfomycin (*fosB2*), beta-lactam (*bla2* and *bla*), carbapenem, and streptothricin (*satA*). Similar findings were also reported in a study conducted in Italy [12]. The results of the WGS analysis showed interesting resistance profiles to some antibiotics; in particular, all strains tested showed genetic resistance to beta-lactams, molecules generally used for the treatment of infections due to *B. anthracis*. However, the presence of predicted antimicrobial resistance genes provides interesting information but would necessarily be combined with phenotypic validation, in order to correctly evaluate antibiotic sensitivity. These biochemical, pathogenic factors and antimicrobial resistance properties of the genome highlight the bacterium’s resilience, adaptability, and the genomic basis of its virulence [52].

Furthermore, based on sequencing analysis, all strains possessed the primary virulence factors: an extracellular capsule of poly-D-glutamate, produced by CapBCAD enzymes located on the virulence plasmid pXO2, lethal toxin (*lef*) and edematous toxin (*cya*), sharing the B-component protective antigen (*pagA*), contained in the virulence plasmid pXO1, confirming the full virulence of the isolated strains.

The present report described the phylogenetic placement of five newly sequenced strains from animal anthrax outbreaks by comparison with publicly available sequencing data. The Ethiopian *B. anthracis* strains were clustered in the A clade at two phylogenetic placements. None of the sequenced genomes in this study are grouped into the *B. anthracis* clades B and C. Two *B. anthracis* strains (BA1 and BA4) belonged to the Trans-Eurasian (TEA) group which is predominantly distributed in large geographic areas, particularly in Eastern Europe [13,18,53] and its presence in both continents could be probably due to commercial exchanges.

In agreement with previous analyses [26], strain A0897 which belongs to the TEA clade (A.Br.161) was isolated from a donkey in 2000, at Kombolcha, Ethiopia, and is related to our investigated two strains (BA1 and BA4) which belong to the same sublineage and also share the same geographic location (Amhara Regional State, northern part of Ethiopia). The strain A0897 was separated by a distance of four SNPs from BA1 and eight SNPs from the BA4 strain. The presence of A.Br.161 (Heroin) clade strains was previously reported in China, Turkey, USA [18], Denmark [54], Norway [55], and Scotland [56]. The clade A.Br.161 (Heroin) has been only reported once in Ethiopia [26], but not in any other part of Africa. According to Price et al. [26], A.Br.161 strains have been linked to an anthrax outbreak among drug users in Europe. All these data, make intriguing the presence of this clade in Ethiopia and not in other African countries, highlighting the necessity of obtaining more genetic data of African *B. anthracis* strains.

Three out of the five sequenced *B. anthracis* strains belonged to the Ancient A Clade, sublineage A.Br.005/006, which is mainly representative of central [57] and southern Africa [58,59].The three strains BA2, BA5, and BA6 belonged to the A.Br.034 subgroup (A.Br.005/006 sublineage), which is common in Africa, Australia, and Europe [9,18] and is included in the Ancient A Clade. These findings strengthen the idea that the fact that historical geographical origin of *B. anthracis* could be Africa [60]. A comparable strain was identified by Eremenko et al. [25], and was isolated in Ukraine in 1957 from a person infected after contact with the contaminated skin of a goat imported from Ethiopia. Pilo and Frey [59] reported that the A.Br.005/006 sublineage is ancestral to other subclades and includes strains that have been isolated mostly from southeastern Africa [61]. The strains in the A.Br.034 subgroup were also isolated from Japan [62], USA, Namibia, and Switzerland [63]. The comparison with genomes of other *B. cereus* group species has not been done, and this could represent a limitation of this study.

However, this study demonstrated the usefulness of WGS as a tool for supporting epidemiological analysis during surveillance activities.

The interpretation of data deriving from the epidemiological data and the characterization of *B. anthracis* genomes present in this territory could be useful for the action of public health institutions in case of possible future outbreaks. To this end, it is necessary to obtain a higher number of sequenced strains in this country and in other African countries, in order to discover and understand the genetic and geographical diversity of *B. anthracis* strains; in this way, it would be possible to have a more defined picture of the epidemiological situation of anthrax in Ethiopia. At the moment, the lack of *B. anthracis* sequences from Ethiopia, deposited in Genbank, could limit the epidemiological monitoring of anthrax.

Thus, this work represents a preliminary stage for a future multi-level assessment of the effectiveness of WGS-based typing during surveillance programs.

Using the data obtained by WGS analyses, it will also be possible to map the risk areas of this country and to define the epidemiological correlations of the circulating strains.

## 5. Conclusions

The finding from this study showed that the genomes of five *B. anthracis* strains isolated from different regions of Ethiopia were placed in two sublineages among the global *B. anthracis* population frame: the Trans-Eurasian (TEA) group and the A.Br.005/006 sublineage (Ancient A Clade). The result of a genotyping method based on the whole genome SNP analysis made it possible to define the current knowledge of the genetic diversity of *B. anthracis* circulating in Ethiopia and to compare it with strains from other parts of the world. As a future step, a comprehensive analysis using a tool like fastANI would also be useful to identify the closest available *B. anthracis* genomes from the NCBI database, ensuring that all relevant comparative sequences are included in the phylogenetic analysis. Further research on the genomes of Ethiopian *B. anthracis* isolates will greatly enhance the current understanding of the phylogenetic structure of the global *B. anthracis* population. This will also broaden the potential for differentiating *B. anthracis* strains and to understand the probable origin and the epidemiological correlations of possible future anthrax outbreaks.

## Figures and Tables

**Table 1:** Source and types of specimens tested to isolate *B. anthracis* strains from different parts of Ethiopia.

ID Strain	Source	Year	Region	Zone	District	Kebele	Sample
BA1	Goat	April 2019	Amhara	South Gondar	Farta	Wowa	Dried meat
BA2	Cow	June 2021	Oromia	West-Shoa	Ada Berga	Bisha Dima	Bone swab
BA3	Goat	December 2021	SNNPR	Gurage	Abeshege	Layegnawgerabana Tachegnawtete	Ear tissue
BA4	Goat	April 2022	Amhara	East Gojjam	Goncha Siso Enese	Angot 028	Skin contaminated with blood
BA5	Cow	November 2022	South West Ethiopia	Kefa	Bonga	02	Ear tissue, swab of oozing blood
BA6	Cow	March 2023	South West Ethiopia	Bench Sheko	South-Bench	Debrwork town	Ear tissue, swab of oozing blood
BA7	Cow	2024	South West Ethiopia	Sheka	Yeki	Yeki	Ear tissue, swab of oozing blood

**Table 2:** Primers and probes used for *B. anthracis* real-time PCR [34].

Target and Primers and Probes	Oligonucleotide Sequence (5′–3′)
PA-Forward:	CGG ATC AAG TAT ATG GGA ATA TAG CAA
PA-Reverse:	CCG GTT TAG TCG TTT CTA ATG GAT
PA-Probe:	FAM-CTC GAA CTG GAG TGA AGT GTT ACC GCA AAT-BHQ1
Cap-Forward:	ACG TAT GGT GTT TCA AGA TTC ATG
Cap-Reverse:	ATT TTC GTC TCA TTC TAC CTC ACC
Cap-Probe:	FAM-CCA CGG AAT TCA AAA ATC TCA AAT GGC AT-BHQ1

**Table 3:** Genomic characteristics of the analyzed *B. anthracis* strains.

Strain	Total Number of Reads	GC %	No of Contigs	Total Length, bp	Average Contig Size	N50, bp	Average Coverage Depth	CDs	tRNA	RGF% *
BA1	1,654,914	35.09	30	5,449,014	1,597,919	601,456	37	5714	77	99.813
BA2	1,593,126	35.09	30	5,449,551	1,769,796	1,162,562	39	5723	77	99.818
BA4	1,995,290	35.10	36	5,448,948	919,145	540,904	48	5716	81	99.784
BA5	2,057,654	36.09	33	5,447,787	1,162,720	918,280	49	5722	81	99.796
BA6	1,644,654	35.09	30	5,449,018	1,768,025	1,198,320	41	5727	78	99.797

* Reference Genome Fraction in %.

## Data Availability

All data generated during this study are included in this published article, and its Supplemental Information Files are publicly available in the NCBI Sequence Read Archive (SRA) repository (Bio Project PRJNA1158707).

## Supplementary Materials

The following supporting information can be downloaded at: https://www.mdpi.com/article/10.3390/pathogens14010039/s1, Figure S1: A minimum-spanning tree of six Ethiopian *B. anthracis* strains, constructed using cgMLST whole genome analysis. The legend specifies the geographical location associated with each sequence, with the Ames Ancestor strain represented by a white circle. Numbers indicate allelic differences between samples. **Table S1**: Metadata of *B. anthracis* strains used in this study.
