# Supplementary material for: Identification of Bacillus anthracis Strains from Animal Cases in Ethiopia and Genetic Characterization by Whole-Genome Sequencing
Source: Pathogens. 2025 Jan 7;14(1):39. doi: 10.3390/pathogens14010039 (PMC11768497; doi:10.3390/pathogens14010039)
Supplement: Supplementary file 1 [file pathogens-14-00039-s001.zip › pathogens-3375372-figure.pdf]

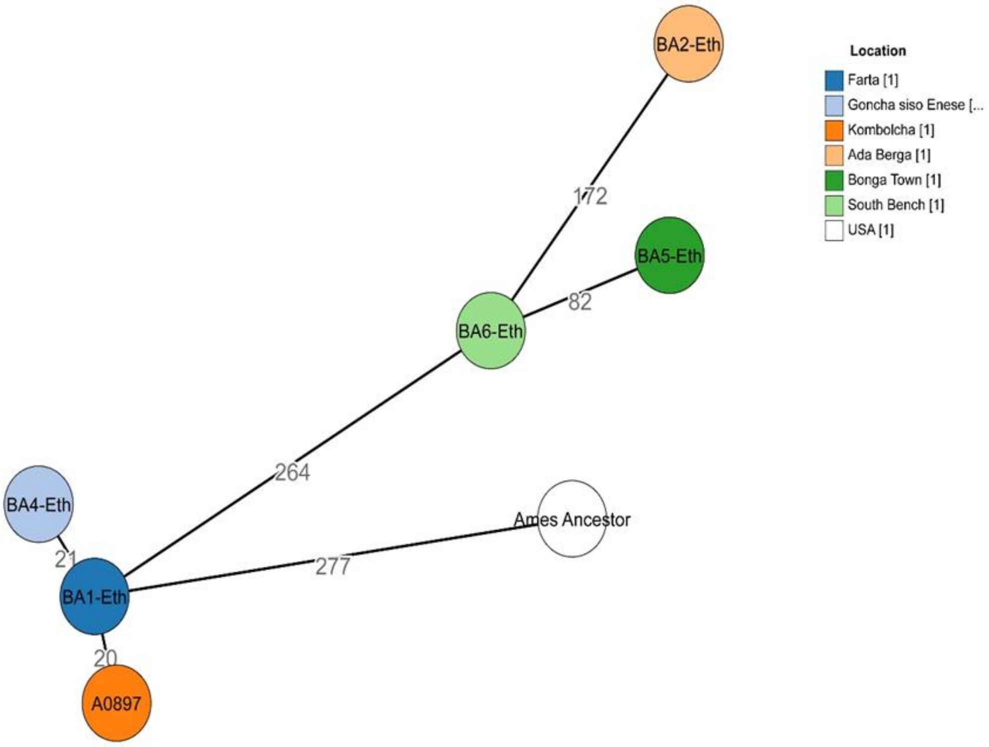

Figure S1. A minimum-spanning tree of six Ethiopian *B. anthracis* strains, constructed using cgMLST whole genome analysis. The legend specifies the geographical location associated with each sequence, with the Ames Ancestor strain represented by a white circle. Numbers indicate allelic differences between samples.
